# Dual Action of Curcumin as an Anti- and Pro-Oxidant from a Biophysical Perspective

**DOI:** 10.3390/antiox12091725

**Published:** 2023-09-06

**Authors:** Agnieszka Wolnicka-Glubisz, Anna Wisniewska-Becker

**Affiliations:** 1 Department of Biophysics and Cancer Biology, Faculty of Biochemistry, Biophysics and Biotechnology, Jagiellonian University, 30-387 Krakow, Poland; 2 Department of Biophysics, Faculty of Biochemistry, Biophysics and Biotechnology, Jagiellonian University, Gronostajowa 7, 30-387 Krakow, Poland

**Keywords:** curcumin, singlet oxygen, melanogenesis, antioxidant, lipid oxidation, oxidative stress

## Abstract

Curcumin, a natural polyphenol widely used as a spice, colorant and food additive, has been shown to have therapeutic effects against different disorders, mostly due to its anti-oxidant properties. Curcumin also reduces the efficiency of melanin synthesis and affects cell membranes. However, curcumin can act as a pro-oxidant when blue light is applied, since upon illumination it can generate singlet oxygen. Our review aims to describe this dual role of curcumin from a biophysical perspective, bearing in mind its concentration, bioavailability-enhancing modifications and membrane interactions, as well as environmental conditions such as light. In low concentrations and without irradiation, curcumin shows positive effects and can be recommended as a beneficial food supplement. On the other hand, when used in excess or irradiated, curcumin can be toxic. Therefore, numerous attempts have been undertaken to test curcumin as a potential photosensitizer in photodynamic therapy (PDT). At that point, we underline that curcumin-based PDT is limited to the treatment of superficial tumors or skin and oral infections due to the weak penetration of blue light. Additionally, we conclude that an increase in curcumin bioavailability through the using nanocarriers, and therefore its concentration, as well as its topical use if skin is exposed to light, may be dangerous.

## 1. Introduction

**Figure 1:**
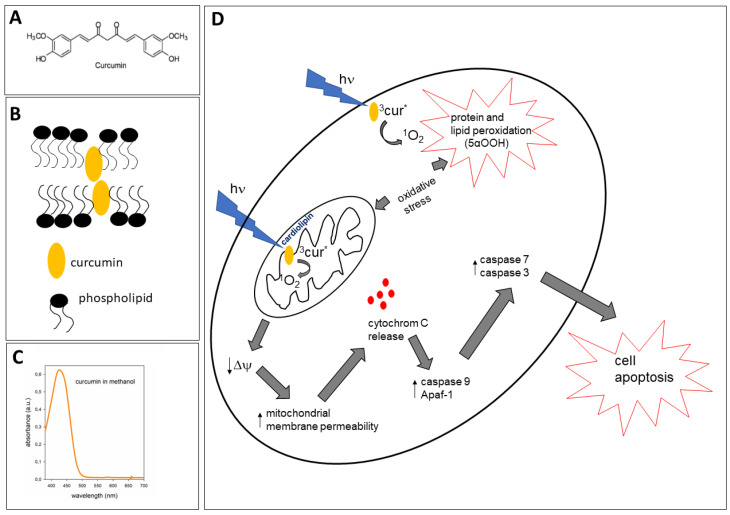
Curcumin chemical structure (**A**), location of curcumin in the membrane (**B**), absorption spectrum of curcumin (10 µM) in methanol in 380–700 nm range (**C**), scheme of phototoxic action of curcumin (**D**). Curcumin localizes in the plasma and inner mitochondrial membranes. Upon blue light absorption, curcumin undergoes activation and eventually forms an excited triple state (^3^cur*). In the presence of oxygen in a Type II photosensitized reaction, due to energy transfer between ^3^cur* and oxygen, singlet oxygen (^1^O_2_) is produced as ROS. As a result of ^1^O_2_-induced oxidative stress, protein and lipid peroxidation occurs, forming, for example, cholesterol hydroperoxides (mainly 5αOOH). In mitochondrial membranes, where mainly cardiolipin is peroxidized, oxidative stress leads to a decrease in membrane potential, an increase in membrane permeability and release of cytochrome c. This activates caspase 9 and Apaf-1. Both caspase 8 and caspase 9 lead to activation of caspase 3 and caspase 7 and consequently to cell apoptosis.

Curcumin is the main active ingredient in turmeric, obtained from the rhizome of *Curcuma longa* [1,2]. This natural polyphenol is a yellow-orange pigment widely used as a spice and food preservative, and also in medicine, especially in Asia [3]. According to many studies, curcumin exhibits various therapeutic properties, among them being anti-cancer, anti-inflammatory, anti-oxidant and wound-healing activities [2,4,5]. The beneficial effects of curcumin have also been shown in cardiovascular, respiratory and neurodegenerative diseases, as well as diabetes and metabolic syndrome [6,7,8]. On the other hand, there are studies showing the toxicity of curcumin to cells and micro-organisms, especially in combination with light [3,9,10,11,12,13]. Phototoxicity may lead to the proapoptotic effects of curcumin, which were observed, for example, in irradiated skin keratinocytes (HaCaT cells) and the human epidermoid carcinoma A431 cell line [14]. The chemical backbone of curcumin [1,7-bis(4-hydroxy-3-methoxyphenyl)-1,6-heptadiene-3,5-dione] (Figure 1A) determines its lipophilic properties and various tautomeric forms [4,8]. The lipophilic properties allow this compound to easily cross cell membranes and act on multiple targets in different cellular pathways, which plays an important role in the pharmacological and biological effects of curcumin on a wide range of diseases [4]. Curcumin can also accumulate in membranes, including plasma and mitochondrial membranes, where it can alter the membrane environment. This may lead to changes in membrane proteins’ properties and functions [15]. Membrane location seems to play a role in both the protective and phototoxic activity of curcumin. In this review, we will present arguments for this dual role of curcumin in biological systems, putting emphasis on the biophysical aspects of its activity, such as interactions with biological membranes and light absorption.

## 2. Curcumin–Membrane Interaction and Its Relevance to Protective and Pro-Oxidant Activity 

The effect of curcumin on membrane properties, as well as its location and orientation within the lipid bilayer, are under debate [16]. On the one hand, there are studies showing a membrane-thinning effect [17,18], fluidization [15,19,20] and an increase in lipid lateral motion [21] in the presence of curcumin. Also, a favoring formation of non-lamellar structures by curcumin has been observed [22]. On the other hand, the ordering of lipids as a result of a curcumin presence in membranes was reported [16,23,24]. There is also no agreement on the location of curcumin in the membrane. Some results suggest that curcumin lies flat on the lipid headgroups, where it forms hydrogen bonds with the lipid molecules [25], or is located near the membrane surface but within the hydrophobic part [26], whereas some others show that curcumin can penetrate deeply into the membrane and intercalate with the lipid tails [15,16,23]. Some studies show that curcumin distribution in the membrane depends on membrane hydration [19] or lipid type [27].

One of the lipids which may prefer to interact with curcumin is cardiolipin. This lipid in *Eucaryota* is present exclusively in the inner mitochondrial membrane, where it constitutes about 20% of all lipids [28]. Because of the tendency to form microdomains of a hexagonal structure, cardiolipin is engaged in the regulation of the functions of proteins involved in respiratory processes, and also in membrane transport and cell division [29,30]. Ben-Zichri et al. [20], using several biophysical techniques, showed in biomimetic and biological mitochondrial membranes that cardiolipin promoted the association and internalization of curcumin into the lipid bilayers. According to the mechanism proposed by the authors, cardiolipin works as a membrane anchor, enhancing the uptake of curcumin. The preferential interaction of curcumin with cardiolipin may lead to an accumulation of curcumin near the cardiolipin headgroups, which in turn may increase the membrane fluidity by loosening the tightly packed phospholipids. This effect on the membrane structure may alter the activity of the proteins involved in respiration processes, and as a result, affect mitochondrial functions. Indeed, curcumin has been shown to modulate different mitochondrial processes. For example, curcumin restored mitochondrial oxidative functions in a mouse model [31] and contributed to the regeneration of mitochondrial functions in the inflamed tissues of obese mice [32]. In tumor cells, curcumin affected mitochondria-induced apoptotic processes [33]. Also, changes in reactive oxygen species (ROS) production in the mitochondrial membrane potential, as well as in the activities of mitochondria-associated proteins, were observed in the presence of curcumin [34,35,36].

The other effects of curcumin on the structural properties of membranes may also have important consequences. The results presented by Duda et al. [16] suggest that curcumin adopts a perpendicular orientation within the membrane. Such an orientation allows curcumin to exert its effect all along the lipid alkyl chains. For instance, it has been shown that curcumin increases the membrane lipid order at all depths within the membrane. Similarly, curcumin increases water penetration not only in the headgroup region but also in the center of the membrane. This means that, on the one hand, an increased lipid order will protect the membrane by making it more resistant to penetration by different compounds, such as oxidants or peptides, but on the other hand, an increased polarity lets more water-soluble free radicals or transition metal ions penetrate into the membrane, which can initiate or re-initiate lipid peroxidation [16]. Membranes of increased polarity may be then more susceptible to oxidative stress. The situation becomes even more complex in the presence of light, which is effectively absorbed by curcumin (λ_max_ of about 420 nm). The process of reactive oxygen species generation by curcumin upon illumination, as well as curcumin’s role as a possible photosensitizer, will be discussed below. Figure 1 presents the scheme of the most probable location of curcumin in the membrane (B) and its absorption spectrum in methanol (C). 

In model membranes, it has been shown that curcumin modulates the formation of lipid raft domains. Tsukamoto et al. observed that curcumin induces the fusion of lipid raft domains at extremely low concentrations through the alteration of the boundary between the ordered and disordered membrane phases [37]. The authors suggested that the boundary-specific action of curcumin may explain the fact that different pharmacological effects of curcumin in the body are expressed by its very low concentration. Our unpublished data show that curcumin prefers to locate not in rafts, but in fluid domains enriched in unsaturated lipids. Depending on the conditions, particularly on the lack or presence of light, such an accumulation of curcumin in regions susceptible to peroxidation can be either beneficiary or harmful.

## 3. Antioxidant Properties of Curcumin 

### 3.1. Curcumin as Reactive Oxygen and Nitrogen Species Scavenger/Quencher

The chemical structure of curcumin (Figure 1A) determines its antioxidant activity, as both the phenolic OH and β-diketone groups of curcumin are involved in neutralizing free radicals, and their relative scavenging capacity depends on the nature of the free radicals [38]. The scavenging of ROS by curcumin is considered a very effective process which leads to considerably less harmful secondary free radicals [39]. In studies using macrophages both *in vitro* and *in vivo* in a rat model, curcumin was shown to have scavenging activity against free radicals such as superoxide radical anion (O_2_^•−^) and nitrite radicals. It was also effective at neutralizing hydrogen peroxide (H_2_O_2_) [40,41]. However, spin trapping studies by Das & Das showed that curcumin is not an effective scavenger of superoxide and hydroxyl radicals [42]. Curcumin has also been shown to act as an antioxidant that breaks the chain at the 3’ position, causing an intramolecular Diels–Alder reaction and neutralizing the lipid radicals [43]. It also inhibits the peroxidation of linoleate, a polyunsaturated fatty acid that can be oxidized to form a fatty acid radical [44].

Another important point in the consideration of the nonselective, systemic action of curcumin is its ability to up-regulate the expression of some genes, and in particular to enhance the production of enzymes involved in biological redox processes (e.g., glutathione synthase GTS, cytochrome P 450 oxidases CYP-450, etc.). On the other hand, curcumin may inhibit other enzymes, such as lipooxygenase and cyclooxygenase (LOX and COX, which are the key enzymes responsible for the transformation of arachidonic acid to prostaglandins), and therefore prevent lipid peroxidation [39]. There are suggestions that this antioxidant activity may be related to the anti-inflammatory effect of curcumin [45,46]. Moreover, Dai et al. showed that curcumin pretreatment significantly reduced furazolidone-induced oxidative stress, leading to a decreased ROS and malondialdehyde formation, an enhancement of the activity of antioxidant enzymes such as superoxide dismutase and catalase, and an increase in glutathione content in human hepatocyte L02 cells [47]. The authors concluded that curcumin protects against furazolidone-induced DNA damage and apoptosis by inhibiting oxidative stress and the mitochondrial pathway. 

In addition to inhibiting lipid peroxidation, curcumin appears to reduce inducible nitric oxide (NO) synthase (iNOS) activity. This enzyme generates large amounts of NO, providing the “oxidative burst” necessary for the defense against pathogens in macrophages. This is possible due to the NO reaction with superoxide anion radicals to form peroxynitrite, which is highly toxic to cells [48]. The effect of curcumin was confirmed in studies on microglia cells (brain macrophage analogs) showing a reduced NO production and the protection of nerve cells from oxidative stress after curcumin treatment [49,50].

Apart from scavenging free radicals and affecting enzyme activity, curcumin may also exert its antioxidant effect by quenching singlet oxygen (^1^O_2_). Singlet oxygen, although not a free radical, is a highly reactive form of oxygen usually produced in photosensitized reactions, in which the excitation energy of a photosensitizer’s triplet state is transferred to molecular oxygen [51]. Das & Das [42] studied the effects of curcumin using Rose Bengal (RB) as a photosensitizer and an electron paramagnetic resonance (EPR) spectroscopy technique with TEMP as a spin trap. RB, a hydrophilic compound, absorbs green light, which is not absorbed by curcumin, and has a high quantum yield of ^1^O_2_ photogeneration (76% upon green light irradiation [52]). Das & Das showed that curcumin is only able to effectively quench ^1^O_2_ at very low concentrations in aqueous systems, whereas it is not an effective scavenger of superoxide and hydroxyl radicals [42]. Nonetheless, Chan et al. in an *in vitro* study, in which human A431 epidermoid carcinoma cells were illuminated in the presence of RB with light emitted by a 120-watt incandescent bulb of undetermined emission [53], suggested that curcumin, by quenching ^1^O_2_, inhibits apoptosis. The authors proved the involvement of ^1^O_2_ in the process by showing that an observed JNK activation, cytochrome c release, caspase activation and subsequent apoptotic biochemical changes were blocked by L-histidine (a known ^1^O_2_ quencher) and α-tocopherol, but not mannitol, which is considered a hydroxyl radical scavenger. 

### 3.2. Inhibitory Effect of Curcumin on Melanogenesis

Melanin is the primary skin pigment synthesized by melanocytes, and to an even greater extent by melanoma cells, in a process called melanogenesis. Melanins, represented by eumelanin, pheomelanin and mixed melanin pigments, are the end products of the complex, multi-step transformations of L-phenylalanine and/or L-tyrosine, with or without L-cysteine and/or glutathione [54,55]. They are commonly considered as versatile photoprotectors, mainly against UV radiation (UVR), and they prevent radiation-induced free-radical damage [54]. However, the presence of melanin can paradoxically lead to the transformation of melanocyte to a malignant state. It was confirmed by Noonan’s studies on mice that melanoma induction via UVA (320–400 nm) required the presence of melanin pigment and was associated with oxidative DNA damage in melanocytes [56].

The regulation of melanin production via the melanocyte-specific melanocortin-1 receptor (MC1R) signaling pathway is a protective mechanism for the skin of living organisms against exposure to UVR [57].

The stimulation of melanogenesis leads to a series of closely related oxidoreductive reactions, causing active melanogenesis, as well as causing melanin to consume oxygen, leading to relative intracellular hypoxia and a potentially mutagenic environment. In addition, the hypoxia-induced reprogramming of the metabolism from predominantly mitochondrial respiration to increased glycolysis in order to maintain ATP levels leads to HIF-1α activation, which, combined with immunosuppressive effects, leads to melanoma progression and resistance to immunotherapy. In addition, the biophysical properties of melanin make melanoma resistant to chemo- and radio-therapy [58]. Therefore, the inhibition of melanogenesis in advanced melanotic melanomas can improve the efficacy of immuno-, chemo- and radio-therapy, and perhaps would itself attenuate melanoma growth [58]. It turns out that one of the factors inhibiting the melanin production process is curcumin. Curcumin and several of its synthetic derivatives have been recognized as tyrosinase inhibitors with interesting therapeutic antimelanogenic activity. Curcumin was found to reduce melanin content and tyrosinase activity in mouse B16 melanoma cells stimulated with α-melanocyte stimulating hormone (α-MSH) [59,60,61] and human melanocyte [62], as well as in a zebrafish embryo model [61]. Tyrosinase is the key rate-limiting enzyme in melanin biosynthesis that catalyzes the hydroxylation of L-tyrosine to L-dihydroxyphenylalanine (L-DOPA) and is expressed by cells of a melanocytic lineage. The intermediates of this process, such as free radicals and highly reactive quinone compounds, can be neutralized by curcumin, so that their potential cytotoxic, genotoxic and mutagenic effects or other regulatory functions will be inhibited. Since its approval by the FDA in 1970, L-DOPA has been used as a prodrug for Parkinson disease (PD), the second most common neurodegenerative disease, as it enhances the intracerebral dopamine concentration [63]. In an animal model of PD in which rotenone was used, curcumin showed additive neuroprotective effects to L-DOPA and rasagiline, and ameliorated the neurodegeneration, DNA fragmentation and motor defects caused by rotenone in mice [64]. It has been shown that the decrease in the activity of tyrosinase under the influence of curcumin is due to a decrease in the expression of melanogenesis-related genes regulated by the MC1R signaling pathway, including MITF, TYR, TRP-1 and TRP-2, through the inhibition of the PI3K/AKT and MAPK/ERK pathways [59,61,62]. As mentioned above, uncontrolled melanogenesis likely plays a key role in melanotic melanoma progression and, along with melanin pigment, influences resistance to radio-, chemo-, phototherapy and immunotherapy [58]. Curcumin’s anti-melanogenetic activity may therefore reverse melanoma progression and restore sensitivity to current therapies, suggesting the potential use of this natural compound as an adjunct to modern melanoma treatments.

However, our study points to an additional mechanism of melanin regulation in melanoma cells treated with curcumin, which is the inhibition of H_2_O_2_ production [65]. Melanogenesis is well known to stimulate intracellular H_2_O_2_ production in melanocytic cells [66]. On the other hand, H_2_O_2_ production affects melanogenesis [67]. Thus, maintaining B16F10 melanoma cells for up to 72 h in a DMEM medium, which contains higher levels of L-tyrosine and L-phenylalanine than RPMI, both of which are essential substrates for melanin synthesis, results in increased levels of melanin, but also H_2_O_2_, in the cells tested. In contrast, the addition of curcumin to the cells results in decreased H_2_O_2_ accumulation and melanin levels [65]. Interestingly, the effect of curcumin on H_2_O_2_ and melanin levels appears to be dependent on H_2_O_2_ concentration. At H_2_O_2_ concentrations greater than 0.3 mM, the inhibitory effect of curcumin begins to prevail, so the effect of curcumin on melanogenesis in B16F10 cells will depend on their condition and how melanogenesis is stimulated [65].

## 4. Proapoptotic Effects of Curcumin 

**Figure 2:**
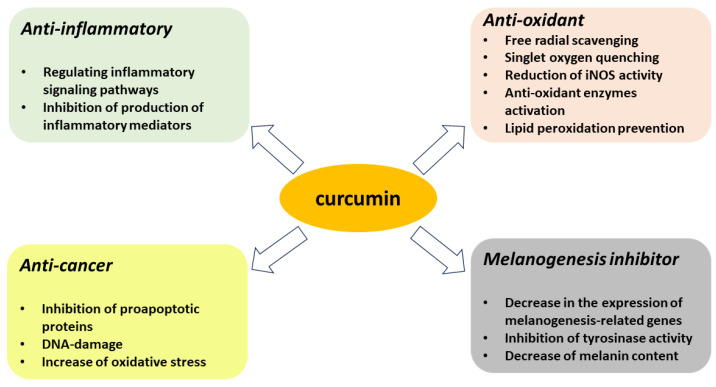
Curcumin protective activities.

Curcumin affects multiple signaling pathways that regulate survival, cell proliferation, apoptosis and tumor suppressor pathways [68]. Based on this, it was concluded that curcumin inhibits the processes of the initiation, progression and metastasis of cancer cells [2]. Indeed, it seems that curcumin’s effect on cancer cells is universal because it has been shown to be effective in breast, lung, prostate, pancreatic, oral and colorectal cancers, as well as in multiple myeloma and squamous cell carcinoma of the head and neck [2]. The regular dietary intake of turmeric by Southwest Asian people is thought to be associated with their lowest incidence of most types of cancer [68]. Curcumin promotes apoptosis by inhibiting the mitochondrial anti-apoptotic proteins BCL-2 and XIAP, which leads to an increased expression of the pro-apoptotic proteins BAX and BAK [68] and changes in the mitochondrial membrane permeability. However, the use of curcumin is limited by its low solubility, rapid metabolism, poor bioavailability, low bioactive absorption and low targeting efficacy, among other factors [2]. To increase efficacy, curcumin can be combined with other anticancer drugs such as 5-fluorouracil, oxaliplatin or gemcitabine [69]. Another approach is to use other methods and forms of curcumin delivery, such as nanoparticles, powder or capsules [2]. The formulation of curcumin into nanoparticles (referred to as nanodiscs, NDs) facilitates its water solubility, and increases curcumin’s binding capacity and targeting potential, which enhance its therapeutic effects [70]. The summary of the most important protective activities of curcumin is presented in Figure 2.

## 5. Pro-Oxidant Properties of Curcumin Induced by Light 

### 5.1. Photogeneration of Singlet Oxygen (^1^O_2_) by Curcumin

One of the first studies on curcumin behavior under illumination was of Chignel et al. [71], in which they focused on the character of curcumin absorption spectra, its fluorescence quantum yield, and the production of ^1^O_2_ and other ROS by curcumin in different solvents. Since then, the spectral and photochemical properties of curcumin in different solvents have been well described, which helps us to understand the biological photoreactivity of curcumin in various cellular microenvironments [3,71,72]. Curcumin absorbs light in the UV–VIS range. An ethanolic solution of curcumin shows three maxima at 220 nm, 262 nm and, in the VIS range, at 424 nm (Figure 1B). Although curcumin’s photoreactivity has been confirmed, and its possible use as a photosensitizer in photodynamic therapy (PDT) has been tested, for example, against micro-organisms or in the case of immunocompromised mice bearing the A431 tumor [1,9,10], there is neither a consensus on the mechanism of this action nor a convincing proof that it can be effective *in vivo*.

Typically, photodynamic activity is related to Type I or Type II photosensitized reactions which lead to the formation of ROS [51]. In Type I, a photosensitizer in the excited state undergoes electron transfer involving either the acquisition or donation of an electron to form the radical cation or radical anion. The radical anion can react with oxygen to produce the superoxide radical anion (O_2_^•−^), and then in consequent reactions, hydrogen peroxide (H_2_O_2_), and eventually the powerful oxidant hydroxyl radical (HO^•^). The Type II process involves an energy transfer from the excited triplet state of a photosensitizer to molecular oxygen, which leads to the formation of ^1^O_2_. Interestingly, most photosensitizers used for PDT are believed to operate via the Type II rather than the Type I mechanism [51]. 

Dahl and co-authors [3] indicated that irradiated curcumin photogenerates ^1^O_2_, superoxide radical anions and possibly H_2_O_2_ in the aprotic environment, and thus mediates oxygen-dependent phototoxicity in rat basophilic leukemia cells. Our recent studies have shown that curcumin under blue light irradiation (438 nm) can generate ^1^O_2_ not only in solvents, but also in liposomes, which were used as a model of cell membranes. In such systems, as well as in cells, the curcumin-generated ^1^O_2_ can diffuse into both the lipid and aqueous phases and cause the oxidation of the proteins and lipids present there. In particular, it was shown that curcumin-generated ^1^O_2_ was the main ROS responsible for the oxidation of cholesterol in liposomes and cells. The application of blue LED light (438 nm) in the presence of 10 µM of curcumin to HaCaT cells showed that the amount of 5α-OOH cholesterol hydroperoxides which are ^1^O_2_-specific [73] was 5.5 times higher than that of free radical-dependent 7α/β-OOH hydroperoxides [13]. The quantum yield of ^1^O_2_ generation by curcumin was estimated to be about 4% [13], which is not particularly high, especially when compared to a known photosensitizer such as Rose Bengal (76% [52]), but seems to be sufficient to induce a photodynamic effect. This is due to the curcumin’s association and accumulation in membranes. 

### 5.2. Phototoxicity and Lipophilicity of Curcumin as a Base for Its Use in PDT

PDT is based on the use of light of a specific wavelength and non-toxic photosensitizers causing a photodynamic effect in order to treat various skin diseases or tumors. The dual-specificity of PDT relies on the accumulation of the photosensitizer in diseased tissue and also on localized light delivery [51]. Due to its hydrophobic nature, curcumin accumulates readily and rapidly (in less than one hour) in cell membranes [3,14] and in mitochondrial membranes, which was shown using confocal microscopy and fluorescence techniques [20]. As a result of such accumulation and blue light irradiation, curcumin-generated ROS (mostly ^1^O_2_) oxidize both lipids and membrane proteins [13] (Figure 1D). Lipid and protein peroxidation was accompanied by a change in the mitochondrial potential and a decrease in the metabolic activity of HaCaT cells, observed immediately after the end of cell irradiation, and also after 24 h [13]. Presumably, depending on the curcumin concentration used, necrosis or apoptosis takes place. The suggested course of action leading to apoptosis is presented in Figure 1D. However, while various concentrations (in the micromolar range) of curcumin are available and can be used in vitro, its bioavailability remains low *in vivo*, limiting its potential use in PDT. The studies of Wozniak et al. [74] on melanoma (MugMel2), squamous cell carcinoma (SCC-25) and normal human keratinocyte (HaCaT) cell lines showed that possible PDT using curcumin can be enhanced by using curcumin encapsulated in hydrogenated soy phosphatidylcholine liposomes. Moreover, as a result of the liposome curcumin-based photodynamic effect, an increased ratio of apoptotic and necrotic cells was observed. The study clearly demonstrated that this form of curcumin decreased malignant cell motility following the treatment. Interestingly, a minimal phototoxic reaction was observed in normal keratinocytes subjected to the same curcumin dose [74]. Therefore, curcumin lipophilicity, which becomes an obstacle in its direct delivery, can come in handy in producing its different formulas, such as liposomes. This would offer extended possibilities for a controlled compound delivery. 

### 5.3. Antimicrobial Photodynamic Activity of Curcumin

The antimicrobial action of curcumin is widely described in a recent review [75]. Here, we focus on its effect in combination with light, since increasing evidence points to the antimicrobial photodynamic activity of curcumin [9,10]. Because bacteria are becoming increasingly resistant to conventional antimicrobial chemotherapy, PDT raises growing interest among scientists and clinicians. Of course, PDT has its limitations, resulting from difficulties with access to light. Generally, PDT against micro-organisms would not be effective in the case of systemic infections but must be focused on the areas where it is relatively easy to apply light. Especially in the case of curcumin, which absorbs blue light (Figure 1C) of low tissue-penetration abilities (0.3–2 mm) [76,77], this limitation has to be considered. However, blue light has high energy, which, absorbed by curcumin, causes the generation of ^1^O_2_, which can diffuse through a micro-organism’s cells and damage different structures. Curcumin-based PDT against micro-organisms makes sense, especially to combat drug-resistant biofilms, since their thickness ranges from 5 to 88 μm, through which even blue light penetrates. Curcumin-based PDT seems to be especially useful in the treatment of the bacteria and fungi responsible for oral and skin infections. In Table 1, we present some examples of curcumin-based PDT against micro-organisms. Lee at al. showed that the viability of *Streptococcus mutans* in the presence of curcumin and *Curcuma xanthorrhiza* extract (CXE), and their mixture, decreases during 405 nm light-emitting diode (LED) irradiation, which can be used to prevent and treat tooth decay using devices that are readily available in clinics [9]. Oral candidiasis, which is the most common opportunistic infection caused by an increased growth and penetration of fungal species in oral tissues [78], can be similarly treated as indicated in studies conducted on various species of *Candida*. Curcumin combined with LED irradiation was effective at inactivating biofilms and cell suspension cultures of clinical isolates of *C. albicans*, *C. glabrata* and *C. tropicalis*, promoting a reduction in the cellular metabolism by 85, 85 and 73%, respectively [79]. The compound was effective at inactivating *C. albicans* present in the tongues of mice with induced oral candidiasis, promoting an approximately 5log10 reduction in cell viability without causing any damage to the animals’ tongue tissues [80]. Independently, Dahl et al. and Dujic et al. confirmed that curcumin can rapidly penetrate cell membranes and accumulate in the cytoplasmic granules located near the nucleus [3,14]. Indeed, Carmello demonstrated the potential of curcumin-assisted photodynamic action to cause DNA damage in *C. albicans* [11]. Widespread candidiasis in immunocompromised patients can cause high mortality [78]. Treatment of *Candida* spp. infections is routinely based on the use of drugs, which can be topical or systemic [81]. However, the use of standard antifungal therapy may be limited due to its toxicity, low efficacy or the resistance of micro-organisms after prolonged exposure to the drug. Curcumin-based PDT is therefore a promising tool. In most of the studies, curcumin was used in a form of solution, with the stock prepared in DMSO or ethanol and then diluted with water (Table 1). However, attempts have been made to increase the effectivity of the treatment against fungi and bacteria by using carriers or special formulas. For example, Perezous et al. and Wang et al. [82,83] showed that the photodynamic activity of curcumin against bacteria can be increased by using it together with silver nanoparticles (Ag NPs) in the core/shell structure nanofiber membrane. In this study, curcumin and Ag NPs were uniformly distributed in the core and shell layers of the fiber membrane, respectively. Ag NPs improve the yield of singlet oxygen generation by curcumin through the metal-enhanced singlet oxygen generation effect [84]. Besides this effect, Ag NPs themselves present antimicrobial activity and are efficient for diagnosis as a contrast in biological images. Thus, the combination of curcumin with Ag NPs (curcumin@Ag) is an interesting multimodal platform involving real-time treatment and diagnosis [85]. The experiments showed that the curcumin@Ag-loaded core/shell nanofiber membrane was very efficient against both *Staphylococcus aureus* and *Escherichia coli*, and its antibacterial rates reached 93.04% and 92.82%, respectively. Interestingly, the antibacterial effectivity of the curcumin@Ag was better than that of the fiber membranes that were single-loaded with curcumin and Ag NPs. Additionally, the curcumin@Ag-loaded core/shell nanofiber membrane exerted a synergistic antibacterial effect on methicillin-resistant *Staphylococcus aureus* [83]. 

Although most of the studies have focused on bacteria and fungi cultures or biofilms, there are also examples of curcumin-based PDT in humans. Leite et al. [86] reported an *in vivo* curcumin application for oral decontamination (salivary micro-organisms). Nine adults were treated with curcumin (30 mg/L) with an incubation time of 5 min and a blue light dose (200 J/cm^2^); nine adults were treated with only with the blue light (200 J/cm^2^); and nine adults were treated only with curcumin (30 mg/L). After the saliva samples were collected, the authors observed that the PDT group showed a significant reduction in microbial viability (up to 5.14 log10- post 1 h) compared with both the blue light and curcumin groups. Another example is the study on 45 adolescent patients performed by Paschoal et al. [87]. The authors reported an *in vivo* evaluation of the antimicrobial and anti-inflammatory properties of curcumin under light activation on the plaque accumulation and gingival bleeding of adolescents under fixed orthodontic treatment. Patients were evaluated using curcumin (1.5 mg/mL) with a fluency of 96 J/cm^2^ and an incubation time of 30 min. After the photodynamic intervention, the dental plaque accumulation via the plaque index (PI) and the gingivitis condition via the gingival bleeding index (GBI), with 1 and 3 months of follow-up, were evaluated. The authors observed that curcumin under photodynamic action was able to control gingivitis after 1 month of follow-up. This outcome was explained by the photodynamic action as well as the anti-inflammatory properties of the curcumin photosensitizer. 

**Table 1:** Examples of photodynamic action of curcumin against various micro-organisms.

Type of Micro-Organisms	Type of Light/Curcumin Formula Used	Ref.
**Bacteria**		
*Streptococcus mutans*	405 nm LED, curcumin and *Curcuma xanthorrhiza* extract	[9]
“oral” bacteria	455 nm LED, curcumin solution	[86]
*Staphylococcus aureus* and *E. coli*	405 nm LED, curcumin@Ag core/shell structure fiber membrane	[83]
*Staphylococcus aureus*	Blue light, curcumin solution	[88]
*Staphylococcus aureus*	Biotable^®^ device 450 nm, curcumin solution	[89]
Methicilin-resistant *Staphylococcus aureus* biofilm	450 nm LED, curcumin solution	[90]
*Propionibacterium acnes*	462 nm LED, curcumin solution	[91]
*Vibrio parahaemolyticus*	470 nm LED, curcumin solution	[92]
*Enterococcus faecalis*	Blue LED, curcumin solution	[93]
*Aggregatibacter actinomycetemcomitans*	420-480 nm LED, curcumin solution	[94]
**Fungi**		
*Candida albicans* and other candidas	455 nm LED, curcumin solution	[79,80,95]
Spores and cells of *Aspergillus niger*, *Aspergillus flavus*, *Penicillium griseofulvum*, *Penicillium chrysogenum*, *Fusarium oxysporum*, *Candida albicans* and *Zygosaccharomyces bailii*	500-Watt Xenon arc lamp, 370–680 nm, curcumin solution (propylene glycol and water)	[96]
*Trichophyton rubrum*	420 nm LED, curcumin solution	[97]

### 5.4. Photodynamic Activity of Curcumin in Skin and Cancer Cells

The skin consists of the epidermis, dermis and subcutaneous tissue, serving as a self-regulating protective organ against environmental influences such as UVR. The body’s homeostasis is regulated by the local neuroendocrine and immune systems through a number of signaling molecules produced by resident and immune cells. The skin’s neuroimmunoendocrine system includes the epidermal neuroendocrine system and interactions with the central systems and organs. Epidermal cells are not only sensitive to neurohormonal regulation, but also produce elements of the hypothalamic–pituitary–adrenal (HPA) or hypothalamic–pituitary–thyroid (HPT) axis, other neuropeptides, biogenic amines, serotonin, melatonin, nitric oxide, opioids, cannabinoids, catecholamines, acetylcholine, steroids, secosteroids, neurotrophins and cytokines. The neuroimmunoendocrine system of the skin can activate central responses with direct homeostatic, metabolic and phenotypic consequences, as described in depth in [98]. The constant exchange of neuroendocrine mediators between the skin and other organs is responsible for maintaining local and global homeostasis, which can be disrupted by stress including UVR, as well as the presence of light-absorbing compounds such as curcumin.

UVR, a component of solar light, has both beneficial and harmful effects on animals and humans. The former includes, for instance, vitamin D3 photoproduction, antimicrobial effects and mood enhancement, whereas the latter includes inflammatory and hyperproliferative disorders of keratinocytes, structural dysfunction of appendages, pigmentation disorders, photoaging and malignancies [98,99]. In addition, the neurotransmitters, hormonal factors, neuropeptides and cytokines released from nerve endings play a key role in the skin’s response to the stress of UVR [98]. The uptake of curcumin by the epidermal cells along with their exposure to UVR may enhance the effect induced by light itself. Indeed, the proapoptotic effect of curcumin was observed in irradiated skin keratinocytes (HaCaT cells) and in the A431 human epidermoid carcinoma cell line [14]. It should be noted that UVA alone causes H_2_O_2_ accumulation in cells, and the use of curcumin in this case showed its antioxidant effect. Moreover, curcumin has a weaker ability to generate H_2_O_2_ than ^1^O_2_ [13]. ^1^O_2_-induced oxidative stress leads to a decrease in mitochondrial membrane potential, an increase in membrane permeability and the release of cytochrome c. This activates caspase 9 and Apaf-1. Both caspase 8 and caspase 9 lead to the activation of caspase 3 and caspase 7, resulting in cell apoptosis (Figure 1D). Interestingly, curcumin combined with visible light mediated tumor growth inhibition in mouse xenograft models of human skin cancer (A431) *in vivo* [1]. However, the study of the effect of curcumin and light, although undertaken on the day of implantation, still did not lead to a complete elimination of the tumor, which does not confirm the high effectiveness of curcumin’s action as a photosensitizer *in vivo*. Despite the not-very-promising results of the *in vivo* studies, curcumin has still been considered as a photosensitizer in PDT against cancer due to its ability to efficiently absorb light and generate ROS. Although, like in the case of bacterial infections, the use of curcumin is limited by the low tissue penetration of blue light [76,77] to treating mostly superficial skin or oral lesions, studies have been undertaken on various cancer cells (not only skin, melanoma or oral, but also kidney, colon and even liver), and the phototoxic effects of curcumin applied in solutions or in the form of nanoparticles in combination with blue light, or light of the entire visible range, have been shown (Table 2). To justify the studies on internal organ cells, one can imagine using a suitable fiber-optic cable with which light can be delivered to tissues through the body’s natural orifices. Such an approach was successfully applied in the case of prostate cancer, however using photosensitizers absorbing red light [100]. PDT can also be vascular-targeted, when photosensitizers accumulate in endothelial cells and a photodynamic effect is imposed not directly on the cancer cells within a tumor, but in the vascular environment [100]. Using such an approach, it could be possible to use even blue light in PDT to treat tumors other than those of the skin, but still of a limited size. Another issue worth mentioning here is the correct use of the term PDT. It refers only to *in vivo* studies, while in vitro-observed effects should rather be described as phototoxic, when a drug and light together induce the effect, or, more specifically, as photodynamic action, when a drug and light in the presence of oxygen induce the effect via the generation of ROS. 

Although the main point of criticism towards curcumin-based PDT is the low penetration of blue light, the studies mentioned in Table 2 also raise some other doubts, especially the study of liver cells in culture [115]; while it is known that most photosensitizers concentrate in the liver, it raises the question of whether this procedure can actually be used to treat liver cancer in situ. Additionally, cell viability was assessed using the MTT assay, which only tests the activity of certain mitochondrial dehydrogenases. Cells were treated with curcumin for 24–48 h, which would not correspond to any effect achievable *in vivo*. In some studies, curcumin was first irradiated and then used to treat cells. Also, clonogenic studies have not been conducted. To make *in vitro* studies meaningful, it is important to apply an appropriate model which can be compared with *in vivo* conditions. Cell monolayers, which are usually obtained in cell cultures, can be easily penetrated by blue light, but *in vivo* such monolayers are not very common. Blue light, however, can pass through several layers of cells (its penetration into tissue is in the range of 0.3–2 mm, and in skin about 1 mm, which is the whole epidermis) [77]; therefore, it can be suggested to use spheroids as a better tumor model. It is known that spheroids cannot be kept in culture for a long time, but it would be interesting, after the treating of such spheroids with curcumin and light, to perform a clonogenic test to assess the effectiveness of the curcumin-imposed photodynamic effect. Spheroids instead of monolayers could be used as a model of cancers such as gastric, bladder, cervical, colon, or even melanoma. In the case of breast, ovarian, kidney, or lung cancer, it is difficult to imagine the effectiveness of curcumin-based PDT due to real difficulties with delivering blue light.

To the best of our knowledge, there are no clinical data available involving curcumin-based PDT against cancer. Because most of the current research on curcumin in combination with light is focused on *in vitro* experiments, and few on animal models, clinical studies are needed to prove its efficacy in PDT. Doing so, it has to be remembered that the therapeutic effect of PDT is determined not only by the bioavailability of curcumin, which can be significantly increased by using different carriers, but also by the depth of penetration of blue light into tissues. Although blue light is a high energy carrier, its ability to penetrate tissues is limited to a maximum depth of 0.3–2 mm [76,77]. The removal of only part of the tumor tissue results in recurrence and may even promote an increase in the metastatic potential of the cancer cells left behind. Thus, blue light is not suitable for use in the PDT of solid tumors, but rather shallow superficial lesions. However, there are some advantages of using curcumin compared to other known photosensitizers, such as its natural origin, ease of acquisition, low price and most importantly, low overall toxicity. Like many other natural compounds from plant sources, curcumin is known for its safety and has been historically consumed by humans for a long period. Also, curcumin’s fluorescence can be used to follow its localization within the organism or cell [20,71]. 

## 6. Conclusions

In this review, we tried to emphasize the double role of curcumin as an anti- and pro-oxidant. From a biophysical point of view, one can be concerned that a compound which can both quench and generate singlet oxygen is neither a perfect photosensitizer nor a good protector against ROS. This would, however, depend on the environmental conditions. In the case of irradiating with blue light, curcumin can act as a photosensitizer, although its photodynamic effects are reduced by its simultaneous ROS quenching. The yield of singlet oxygen generation, which was shown to be at about 4%, is therefore an effective one, since without the quenching it could be higher. The antioxidant action of curcumin may prevail when samples are irradiated in the presence of another photosensitizer, such as Rose Bengal or porphyrins, which absorb light of longer wavelengths. Such a swap from anti- to pro-oxidant features, as well as the simultaneous generation and quenching of ^1^O_2_ by a single species, is a known phenomenon. For example, it was shown for melanin [128] and carotenoids [129,130,131]. Another biophysical aspect which raises doubts is the weak tissue penetration of the light absorbed by curcumin. Curcumin may not be a perfect photosensitizer, not because it is inefficient at ROS production, but because its use in PDT has to be limited to the depth of 0.3–2 mm of the tissue [76,77]. It still, however, can be useful in PDT against micro-organisms. On the other hand, the topical application of curcumin in the form of creams or sunscreens [132] should not be advised. In the absence of light, and in low concentrations, curcumin shows various positive effects stemming from its antioxidant abilities, and can be recommended as a beneficial food supplement. 

As we tried to show in our review, curcumin, due to its natural origin, ease of acquisition and widespread consumption, has been studied thoroughly and in different aspects. Epidemiological observations indicate, for example, that the regular consumption of turmeric by the Asian population may promote a decrease in the incidence of various types of cancer in this population. Curcumin’s spectrum of action is very broad, which makes its usage tempting not only in traditional Chinese medicine but also in modern therapies. Curcumin has a relatively poor bioavailability, which, at first glance, may be a disadvantage. Hence, attempts are made to increase its bioavailability by using different carriers such as liposomes, nanoparticles, etc. However, it can be suggested that this poor bioavailability is the key to curcumin’s health-promoting effects, because, as mentioned above, an excessive concentration in cells or excessive exposure to UVor blue light can have the opposite, even undesirable, effects on healthy tissues. The use of high concentrations of curcumin makes sense only in anti-cancer or anti-microbial therapies such as PDT, when selectivity can be achieved by using nanocarriers or applying light that reaches only the affected areas. However, one has to underline, once again, that curcumin’s efficacy as a photosensitizing agent has serious limitations due to its inadequate absorbance spectrum, its application only to very small tumor volumes *in vivo*, and the lack of any convincing information on *in vivo* efficacy, as most of the current research literature includes only *in vitro* examples.

## Figures and Tables

**Table 2:** Photodynamic action of curcumin against various cancer cells in vitro.

Type of Cancer Cells	Type of Light and Formula Used	Ref
**Oral cancer**		
HN cells	VIS, 5500 lx, UVA, curcumin solution	[101]
**Skin cancer cells**		
A431/xenograft	VIS, 5500 lx, UVA, curcumin solution	[1]
A431	VIS, curcumin solution	
A431	VIS/PEGylated lipid nanocarrier in vitro	[102]
SCC	380–550 nm, Lip-cur	[74]
**Melanoma**		
G-361, A375	VIS, 5500 lx, UVA, curcumin solution	[103]
A375	combination 630 nm + 405 nm polarized blue light, solution DMSO	[104]
A735, C32		[105]
MugMel2	VIS (380–550 nm), Lip-cur	[74]
**Bladder cancer**		
RT112,UMUC3, TCCSUP	VIS, 5500 lx, curcumin solution	[106]
RT112,UMUC3, TCCSUP	VIS, 5500 lx, curcumin solution	[107]
**Colon cancer**		
SW620, HT29	470 nm, curcumin solution	[108]
CaCo2	5-ALA + 635 nm diode laser system/solution DMSO	[109]
CaCo2	424 nm, CAgNPs	[85]
mouse colorectal-CT26	450 nm, F127-curcumin micelles	[110]
**Prostate cancer**		
PC3	5-ALA + 635 nm diode laser system/solution DMSO	[109]
LNCaP	430 nm LED, curcumin solution	[111]
**Kidney cancer**		
A498, Caki1, KTCTL-26	VIS, 5500 lx, curcumin solution	[112]
A498, Caki1, KTCTL-26	VIS, 5500 lx, curcumin solution	[113]
**Liver cancer**		
SMMC-7721	430 nm, curcumin solution	[114]
HuH6, HepT1,Hep-G2, HC-AFW1	390–440 nm, curcumin solution	[115]
**Cervical cancer**		
HeLa	VIS (400–700 nm), curcumin solution	[116]
Me180	445 nm laser, Cur-LDH	[117]
SiHa, CasKi	447 nm LED, nano-emulsion	[118]
**Lung cancer**		
A549	430 nm LED, Cur-SLN	[119]
A549	457 nm LED, PLGA nanoparticles	[120]
**Ovarian cancer**		
SK-OV3	457 nm, 620 nm LED), Cur-NP	[121]
**Breast cancer**		
MCF-7	430 nm, Cur-NLCs	[122]
MCF-7	440 nm LED, nano-emulsion	[123]
MDA-MB-231	curcumin-LDH nanoparticles	[124]
mouse 4T1	Cur NDs	[125]
mouse 4T1	Photothermal/Au-Cur nanostructure	[126]
**Gastric cancer**		
MKN45	460 nm LED, nano-encapsulated curcumin with EGF	[127]

VIS—visible light, Lip-cur—liposomal curcumin, Cur-SLN—curcumin–solid–liquid nanomaterial, Cur-AgNPs—curcumin conjugated with the silver nanoparticles, Cur-NLCs—curcumin nano-lipid carriers, EGF—epidermal growth factor, Cur-NP, Cur NDs—curcumin in nanoparticles, Au-Cur—gold–curcumin nanostructure.

## Data Availability

Not applicable.

## References

[1] Dujic J., Kippenberger S., Ramirez-Bosca A., Diaz-Alperi J., Bereiter-Hahn J., Kaufmann R., Bernd A., Hofmann M. (2009). Curcumin in Combination with Visible Light Inhibits Tumor Growth in a Xenograft Tumor Model. Int. J. Cancer.

[2] Shanmugam M., Rane G., Kanchi M., Arfuso F., Chinnathambi A., Zayed M., Alharbi S., Tan B., Kumar A., Sethi G. (2015). The Multifaceted Role of Curcumin in Cancer Prevention and Treatment. Molecules.

[3] Dahll T.A., Bilski P., Reszka K.J., Chignell C.F. (1994). Photocytotoxicity of Curcumin. Photochem. Photobiol..

[4] Panahi Y., Fazlolahzadeh O., Atkin S.L., Majeed M., Butler A.E., Johnston T.P., Sahebkar A. (2019). Evidence of Curcumin and Curcumin Analogue Effects in Skin Diseases: A Narrative Review. J. Cell Physiol..

[5] Wu Y., Zhang P., Yang H., Ge Y., Xin Y. (2017). Effects of Demethoxycurcumin on the Viability and Apoptosis of Skin Cancer Cells. Mol. Med. Rep..

[6] Barbalho S.M., Sousa Gonzaga H.F., Souza G.A., Alvares Goulart R., Sousa Gonzaga M.L., Alvarez Rezende B. (2021). Dermatological Effects of *Curcuma* Species: A Systematic Review. Clin. Exp. Dermatol..

[7] Monroy A., Lithgow G.J., Alavez S. (2013). Curcumin and Neurodegenerative Diseases. BioFactors.

[8] Nabavi S., Thiagarajan R., Rastrelli L., Daglia M., Sobarzo-Sanchez E., Alinezhad H., Nabavi S. (2015). Curcumin: A Natural Product for Diabetes and Its Complications. Curr. Top. Med. Chem..

[9] Lee H.-J., Kang S.-M., Jeong S.-H., Chung K.-H., Kim B.-I. (2017). Antibacterial Photodynamic Therapy with Curcumin and *Curcuma xanthorrhiza* Extract against *Streptococcus mutans*. Photodiagnosis Photodyn. Ther..

[10] Bhavya M.L., Umesh Hebbar H. (2019). Efficacy of Blue LED in Microbial Inactivation: Effect of Photosensitization and Process Parameters. Int. J. Food Microbiol..

[11] Carmello J.C., Pavarina A.C., Oliveira R., Johansson B. (2015). Genotoxic Effect of Photodynamic Therapy Mediated by Curcumin on Candida Albicans. FEMS Yeast Res..

[12] Cozzolino M., Delcanale P., Montali C., Tognolini M., Giorgio C., Corrado M., Cavanna L., Bianchini P., Diaspro A., Abbruzzetti S. (2019). Enhanced Photosensitizing Properties of Protein Bound Curcumin. Life Sci..

[13] Wolnicka-Glubisz A., Olchawa M., Duda M., Pabisz P., Wisniewska-Becker A. (2023). The Role of Singlet Oxygen in Photoreactivity and Phototoxicity of Curcumin. Photochem. Photobiol..

[14] Dujic J., Kippenberger S., Hoffmann S., Ramirez-Bosca A., Miquel J., Diaz-Alperi J., Bereiter-Hahn J., Kaufmann R., Bernd A. (2007). Low Concentrations of Curcumin Induce Growth Arrest and Apoptosis in Skin Keratinocytes Only in Combination with UVA or Visible Light. J. Investig. Dermatol..

[15] Ingolfsson H.I., Koeppe R.E., Andersen O.S. (2007). Curcumin Is a Modulator of Bilayer Material Properties. Biochemistry.

[16] Duda M., Cygan K., Wisniewska-Becker A. (2020). Effects of Curcumin on Lipid Membranes: An EPR Spin-Label Study. Cell Biochem. Biophys..

[17] Hung W.-C., Chen F.-Y., Lee C.-C., Sun Y., Lee M.-T., Huang H.W. (2008). Membrane-Thinning Effect of Curcumin. Biophys. J..

[18] Sun Y., Lee C.-C., Hung W.-C., Chen F.-Y., Lee M.-T., Huang H.W. (2008). The Bound States of Amphipathic Drugs in Lipid Bilayers: Study of Curcumin. Biophys. J..

[19] Alsop R.J., Dhaliwal A., Rheinstädter M.C. (2017). Curcumin Protects Membranes through a Carpet or Insertion Model Depending on Hydration. Langmuir.

[20] Ben-Zichri S., Kolusheva S., Danilenko M., Ossikbayeva S., Stabbert W.J., Poggio J.L., Stein D.E., Orynbayeva Z., Jelinek R. (2019). Cardiolipin Mediates Curcumin Interactions with Mitochondrial Membranes. Biochim. Biophys. Acta BBA—Biomembr..

[21] Sharma V.K., Gupta J., Srinivasan H., Bhatt H., García Sakai V., Mitra S. (2022). Curcumin Accelerates the Lateral Motion of DPPC Membranes. Langmuir.

[22] Pérez-Lara A., Ausili A., Aranda F.J., de Godos A., Torrecillas A., Corbalán-García S., Gómez-Fernández J.C. (2010). Curcumin Disorders 1,2-Dipalmitoyl-*Sn*-Glycero-3-Phosphocholine Membranes and Favors the Formation of Nonlamellar Structures by 1,2-Dielaidoyl-*Sn*-Glycero-3-Phosphoethanolamine. J. Phys. Chem. B.

[23] Barry J., Fritz M., Brender J.R., Smith P.E.S., Lee D.-K., Ramamoorthy A. (2009). Determining the Effects of Lipophilic Drugs on Membrane Structure by Solid-State NMR Spectroscopy: The Case of the Antioxidant Curcumin. J. Am. Chem. Soc..

[24] Kotenkov S.A., Gnezdilov O.I., Khaliullina A.V., Antzutkin O.N., Gimatdinov R.S., Filippov A.V. (2019). Effect of Cholesterol and Curcumin on Ordering of DMPC Bilayers. Appl. Magn. Reason..

[25] Varshney G.K., Kintali S.R., Gupta P.K., Das K. (2016). Effect of Bilayer Partitioning of Curcumin on the Adsorption and Transport of a Cationic Dye Across POPG Liposomes Probed by Second-Harmonic Spectroscopy. Langmuir.

[26] Ausili A., Gómez-Murcia V., Candel A.M., Beltrán A., Torrecillas A., He L., Jiang Y., Zhang S., Teruel J.A., Gómez-Fernández J.C. (2021). A Comparison of the Location in Membranes of Curcumin and Curcumin-Derived Bivalent Compounds with Potential Neuroprotective Capacity for Alzheimer’s Disease. Colloids Surf. B. Biointerfaces.

[27] Lyu Y., Xiang N., Mondal J., Zhu X., Narsimhan G. (2018). Characterization of Interactions between Curcumin and Different Types of Lipid Bilayers by Molecular Dynamics Simulation. J. Phys. Chem. B..

[28] Schlame M., Brody S., Hostetler K.Y. (1993). Mitochondrial Cardiolipin in Diverse Eukaryotes. Comparison of Biosynthetic Reactions and Molecular Acyl Species. Eur. J. Biochem..

[29] Jouhet J. (2013). Importance of the Hexagonal Lipid Phase in Biological Membrane Organization. Front. Plant Sci..

[30] Mileykovskaya E., Dowhan W. (2009). Cardiolipin Membrane Domains in Prokaryotes and Eukaryotes. Biochim. Biophys. Acta BBA—Biomembr..

[31] Soto-Urquieta M.G., López-Briones S., Pérez-Vázquez V., Saavedra-Molina A., González-Hernández G.A., Ramírez-Emiliano J. (2014). Curcumin Restores Mitochondrial Functions and Decreases Lipid Peroxidation in Liver and Kidneys of Diabetic Db/Db Mice. Biol. Res..

[32] Kuo J.-J., Chang H.-H., Tsai T.-H., Lee T.-Y. (2012). Positive Effect of Curcumin on Inflammation and Mitochondrial Dysfunction in Obese Mice with Liver Steatosis. Int. J. Mol. Med..

[33] Pesakhov S., Khanin M., Studzinski G.P., Danilenko M. (2010). Distinct Combinatorial Effects of the Plant Polyphenols Curcumin, Carnosic Acid, and Silibinin on Proliferation and Apoptosis in Acute Myeloid Leukemia Cells. Nutr. Cancer.

[34] Sen S., Sharma H., Singh N. (2005). Curcumin Enhances Vinorelbine Mediated Apoptosis in NSCLC Cells by the Mitochondrial Pathway. Biochem. Biophys. Res. Commun..

[35] Wang J., Qi L., Zheng S., Wu T. (2009). Curcumin Induces Apoptosis through the Mitochondria-Mediated Apoptotic Pathway in HT-29 Cells. J. Zhejiang Univ. Sci. B..

[36] Ramamoorthy H., Abraham P., Isaac B. (2014). Mitochondrial Dysfunction and Electron Transport Chain Complex Defect in a Rat Model of Tenofovir Disoproxil Fumarate Nephrotoxicity. J. Biochem. Mol. Toxicol..

[37] Tsukamoto M., Kuroda K., Ramamoorthy A., Yasuhara K. (2014). Modulation of Raft Domains in a Lipid Bilayer by Boundary-Active Curcumin. Chem. Commun..

[38] Singh U., Barik A., Singh B.G., Priyadarsini K.I. (2011). Reactions of Reactive Oxygen Species (ROS) with Curcumin Analogues: Structure–Activity Relationship. Free Radic. Res..

[39] Grynkiewicz G., Ślifirski P. (2012). Curcumin and Curcuminoids in Quest for Medicinal Status. Acta Biochim. Pol..

[40] Joe B., Vijaykumar M., Lokesh B.R. (2004). Biological Properties of Curcumin-Cellular and Molecular Mechanisms of Action. Crit. Rev. Food Sci. Nutr..

[41] Joe B., Lokesh B.R. (1994). Role of Capsaicin, Curcumin and Dietary *n* − 3 Fatty Acids in Lowering the Generation of Reactive Oxygen Species in Rat Peritoneal Macrophages. Biochim. Biophys. Acta BBA—Mol. Cell Res..

[42] Das K.C., Das C.K. (2002). Curcumin (Diferuloylmethane), a Singlet Oxygen (^1^O_2_) Quencher. Biochem. Biophys. Res. Commun..

[43] Sreejayan, Rao M.N.A. (2011). Curcuminoids as Potent Inhibitors of Lipid Peroxidation. J. Pharm. Pharmacol..

[44] Masuda T., Maekawa T., Hidaka K., Bando H., Takeda Y., Yamaguchi H. (2001). Chemical Studies on Antioxidant Mechanism of Curcumin: Analysis of Oxidative Coupling Products from Curcumin and Linoleate. J. Agric. Food Chem..

[45] Aggarwal B.B., Harikumar K.B. (2009). Potential Therapeutic Effects of Curcumin, the Anti-Inflammatory Agent, against Neurodegenerative, Cardiovascular, Pulmonary, Metabolic, Autoimmune and Neoplastic Diseases. Int. J. Biochem. Cell Biol..

[46] Shehzad A., Wahid F., Lee Y.S. (2010). Curcumin in Cancer Chemoprevention: Molecular Targets, Pharmacokinetics, Bioavailability, and Clinical Trials. Arch. Pharm..

[47] Dai C., Li D., Gong L., Xiao X., Tang S. (2016). Curcumin Ameliorates Furazolidone-Induced DNA Damage and Apoptosis in Human Hepatocyte L02 Cells by Inhibiting ROS Production and Mitochondrial Pathway. Molecules.

[48] Pacher P., Beckman J.S., Liaudet L. (2007). Nitric Oxide and Peroxynitrite in Health and Disease. Physiol. Rev..

[49] Jung K.K., Lee H.S., Cho J.Y., Shin W.C., Rhee M.H., Kim T.G., Kang J.H., Kim S.H., Hong S., Kang S.Y. (2006). Inhibitory Effect of Curcumin on Nitric Oxide Production from Lipopolysaccharide-Activated Primary Microglia. Life Sci..

[50] He L., Chen H., Qian L., Chen G., Buzby J.S. (2010). Curcumin Protects Pre-Oligodendrocytes from Activated Microglia in Vitro and in Vivo. Brain Res..

[51] Abrahamse H., Hamblin M.R. (2016). New Photosensitizers for Photodynamic Therapy. Biochem. J..

[52] Wilkinson F., Helman W.P., Ross A.B. (1993). Quantum Yields for the Photosensitized Formation of the Lowest Electronically Excited Singlet State of Molecular Oxygen in Solution. J. Phys. Chem. Ref. Data.

[53] Chan W.-H., Wu H.-J. (2004). Anti-Apoptotic Effects of Curcumin on Photosensitized Human Epidermal Carcinoma A431 Cells. J. Cell. Biochem..

[54] Slominski A., Tobin D.J., Shibahara S., Wortsman J. (2004). Melanin Pigmentation in Mammalian Skin and Its Hormonal Regulation. Physiol. Rev..

[55] Schallreuter K.U., Kothari S., Chavan B., Spencer J.D. (2008). Regulation of Melanogenesis—Controversies and New Concepts. Exp. Dermatol..

[56] Noonan F.P., Zaidi M.R., Wolnicka-Glubisz A., Anver M.R., Bahn J., Wielgus A., Cadet J., Douki T., Mouret S., Tucker M.A. (2012). Melanoma Induction by Ultraviolet A but Not Ultraviolet B Radiation Requires Melanin Pigment. Nat. Commun..

[57] Abdel-Malek Z.A., Knittel J., Kadekaro A.L., Swope V.B., Starner R. (2008). The Melanocortin 1 Receptor and the UV Response of Human Melanocytes—A Shift in Paradigm. Photochem. Photobiol..

[58] Slominski R.M., Sarna T., Płonka P.M., Raman C., Brożyna A.A., Slominski A.T. (2022). Melanoma, Melanin, and Melanogenesis: The Yin and Yang Relationship. Front. Oncol..

[59] Lee J.-H., Jang J.-Y., Park C., Kim B.-W., Choi Y.-H., Choi B.-T. (2010). Curcumin Suppresses α-Melanocyte Stimulating Hormone-Stimulated Melanogenesis in B16F10 Cells. Int. J. Mol. Med..

[60] Park S.Y., Jin M.L., Kim Y.H., Kim Y., Lee S.-J. (2011). Aromatic-Turmerone Inhibits α-MSH and IBMX-Induced Melanogenesis by Inactivating CREB and MITF Signaling Pathways. Arch. Dermatol. Res..

[61] Jeon H.-J., Kim K., Kim C., Lee S.-E. (2023). Antimelanogenic Effects of Curcumin and Its Dimethoxy Derivatives: Mechanistic Investigation Using B16F10 Melanoma Cells and Zebrafish (Danio Rerio) Embryos. Foods.

[62] Tu C.-X., Lin M., Lu S.-S., Qi X.-Y., Zhang R.-X., Zhang Y.-Y. (2012). Curcumin Inhibits Melanogenesis in Human Melanocytes. Phytother. Res..

[63] Nebrisi E. (2021). El Neuroprotective Activities of Curcumin in Parkinson’s Disease: A Review of the Literature. Int. J. Mol. Sci..

[64] El-Shamarka M.E.-S., Abdel-Salam O.M., Shafee N., Zeidan H.M. (2023). Curcumin Modulation of L-Dopa and Rasagiline-Induced Neuroprotection in Rotenone Model of Parkinson’s Disease. Iran. J. Basic Med. Sci..

[65] Wolnicka-Glubisz A., Nogal K., Żądło A., Płonka P.M. (2015). Curcumin Does Not Switch Melanin Synthesis towards Pheomelanin in B16F10 Cells. Arch. Dermatol. Res..

[66] Mastore M., Kohler L., Nappi A.J. (2005). Production and Utilization of Hydrogen Peroxide Associated with Melanogenesis and Tyrosinase-Mediated Oxidations of DOPA and Dopamine. FEBS J..

[67] Jiménez-Cervantes C., Martínez-Esparza M., Pérez C., Daum N., Solano F., García-Borrón J.C. (2001). Inhibition of Melanogenesis in Response to Oxidative Stress: Transient Downregulation of Melanocyte Differentiation Markers and Possible Involvement of Microphthalmia Transcription Factor. J. Cell Sci..

[68] Ravindran J., Prasad S., Aggarwal B.B. (2009). Curcumin and Cancer Cells: How Many Ways Can Curry Kill Tumor Cells Selectively?. AAPS J..

[69] Kanai M. (2014). Therapeutic Applications of Curcumin for Patients with Pancreatic Cancer. World J. Gastroenterol..

[70] Crosby N.M., Ghosh M., Su B., Beckstead J.A., Kamei A., Simonsen J.B., Luo B., Gordon L.I., Forte T.M., Ryan R.O. (2015). Anti-CD20 Single Chain Variable Antibody Fragment–Apolipoprotein A-I Chimera Containing Nanodisks Promote Targeted Bioactive Agent Delivery to CD20-Positive Lymphomas. Biochem. Cell Biol..

[71] Chignell C.F., Bilski P., Reszka K.J., Motten A.G., Sik R.H., Dahl T.A. (1994). Spectral and Photochemical Properties of Curcumin. Photochem. Photobiol..

[72] Khopde S.M., Indira Priyadarsini K., Palit D.K., Mukherjee T. (2000). Effect of Solvent on the Excited-State Photophysical Properties of Curcumin. Photochem. Photobiol..

[73] Korytowski W., Bachowski G.J., Girotti A.W. (1993). Analysis of Cholesterol and Phospholipid Hydroperoxides by High-Performance Liquid Chromatography with Mercury Drop Electrochemical Detection. Anal. Biochem..

[74] Woźniak M., Nowak M., Lazebna A., Więcek K., Jabłońska I., Szpadel K., Grzeszczak A., Gubernator J., Ziółkowski P. (2021). The Comparison of In Vitro Photosensitizing Efficacy of Curcumin-Loaded Liposomes Following Photodynamic Therapy on Melanoma MUG-Mel2, Squamous Cell Carcinoma SCC-25, and Normal Keratinocyte HaCaT Cells. Pharmaceuticals.

[75] Dai C., Lin J., Li H., Shen Z., Wang Y., Velkov T., Shen J. (2022). The Natural Product Curcumin as an Antibacterial Agent: Current Achievements and Problems. Antioxidants.

[76] Barolet D. (2008). Light-Emitting Diodes (LEDs) in Dermatology. Semin. Cutan. Med. Surg..

[77] Ash C., Dubec M., Donne K., Bashford T. (2017). Effect of Wavelength and Beam Width on Penetration in Light-Tissue Interaction Using Computational Methods. Lasers Med. Sci..

[78] Pfaller M.A., Diekema D.J. (2007). Epidemiology of Invasive Candidiasis: A Persistent Public Health Problem. Clin. Microbiol. Rev..

[79] Dovigo L.N., Pavarina A.C., Carmello J.C., Machado A.L., Brunetti I.L., Bagnato V.S. (2011). Susceptibility of Clinical Isolates of *Candida* to Photodynamic Effects of Curcumin. Lasers Surg. Med..

[80] Dovigo L.N., Carmello J.C., de Souza Costa C.A., Vergani C.E., Brunetti I.L., Bagnato V.S., Pavarina A.C. (2013). Curcumin-Mediated Photodynamic Inactivation of *Candida albicans* in a Murine Model of Oral Candidiasis. Med. Mycol..

[81] Pappas P.G., Kauffman C.A., Andes D., Benjamin D.K., Calandra T.F., Edwards J.E., Filler S.G., Fisher J.F., Kullberg B.-J., Zeichner L.O. (2009). Clinical Practice Guidelines for the Management Candidiasis: 2009 Update by the Infectious Diseases Society of America. Clin. Infect. Dis..

[82] Perezous L.F., Flaitz C.M., Goldschmidt M.E., Engelmeier R.L. (2005). Colonization of *Candida* Species in Denture Wearers with Emphasis on HIV Infection: A Literature Review. J. Prosthet. Dent..

[83] Wang Q., Liu S., Lu W., Zhang P. (2022). Fabrication of Curcumin@Ag Loaded Core/Shell Nanofiber Membrane and Its Synergistic Antibacterial Properties. Front. Chem..

[84] Zhang Y., Aslan K., Previte M.J.R., Geddes C.D. (2008). Plasmonic Engineering of Singlet Oxygen Generation. Proc. Natl. Acad. Sci. USA.

[85] de Freitas C.F., Kimura E., Rubira A.F., Muniz E.C. (2020). Curcumin and Silver Nanoparticles Carried out from Polysaccharide-Based Hydrogels Improved the Photodynamic Properties of Curcumin through Metal-Enhanced Singlet Oxygen Effect. Mater. Sci. Eng. C..

[86] Leite D.P.V., Paolillo F.R., Parmesano T.N., Fontana C.R., Bagnato V.S. (2014). Effects of Photodynamic Therapy with Blue Light and Curcumin as Mouth Rinse for Oral Disinfection: A Randomized Controlled Trial. Photomed. Laser Surg..

[87] Paschoal M.A., Moura C.M.Z., Jeremias F., Souza J.F., Bagnato V.S., Giusti J.S.M., Santos-Pinto L. (2015). Longitudinal Effect of Curcumin-Photodynamic Antimicrobial Chemotherapy in Adolescents during Fixed Orthodontic Treatment: A Single-Blind Randomized Clinical Trial Study. Lasers Med. Sci..

[88] Jiang Y., Leung A.W., Hua H., Rao X., Xu C. (2014). Photodynamic Action of LED-Activated Curcumin against *Staphylococcus aureus* Involving Intracellular ROS Increase and Membrane Damage. Int. J. Photoenergy.

[89] Dias L.D., Aguiar A.S.N., de Melo N.J., Inada N.M., Borges L.L., de Aquino G.L.B., Camargo A.J., Bagnato V.S., Napolitano H.B. (2023). Structural Basis of Antibacterial Photodynamic Action of Curcumin against *S. aureus*. Photodiagnosis Photodyn. Ther..

[90] de Paula Ribeiro I., Pinto J.G., Souza B.M.N., Miñán A.G., Ferreira-Strixino J. (2022). Antimicrobial Photodynamic Therapy with Curcumin on Methicillin-Resistant *Staphylococcus aureus* biofilm. Photodiagnosis Photodyn. Ther..

[91] Yang M.-Y., Chang K.-C., Chen L.-Y., Hu A. (2018). Low-Dose Blue Light Irradiation Enhances the Antimicrobial Activities of Curcumin against *Propionibacterium acnes*. J. Photochem. Photobiol. B.

[92] Wu J., Mou H., Xue C., Leung A.W., Xu C., Tang Q.-J. (2016). Photodynamic Effect of Curcumin on *Vibrio parahaemolyticus*. Photodiagnosis Photodyn. Ther..

[93] da Frota M.F., Guerreiro-Tanomaru J.M., Tanomaru-Filho M., Bagnato V.S., Espir C.G., Berbert F.L.C.V. (2015). Photodynamic Therapy in Root Canals Contaminated with *Enterococcus faecalis* Using Curcumin as Photosensitizer. Lasers Med. Sci..

[94] Najafi S., Khayamzadeh M., Paknejad M., Poursepanj G., Kharazi Fard M.J., Bahador A. (2016). An In Vitro Comparison of Antimicrobial Effects of Curcumin-Based Photodynamic Therapy and Chlorhexidine, on *Aggregatibacter actinomycetemcomitans*. J. Lasers Med. Sci..

[95] Dovigo L.N., Pavarina A.C., Ribeiro A.P.D., Brunetti I.L., Costa C.A.d.S., Jacomassi D.P., Bagnato V.S., Kurachi C. (2011). Investigation of the Photodynamic Effects of Curcumin Against *Candida albicans*. Photochem. Photobiol..

[96] Al-Asmari F., Mereddy R., Sultanbawa Y. (2017). A Novel Photosensitization Treatment for the Inactivation of Fungal Spores and Cells Mediated by Curcumin. J. Photochem. Photobiol. B..

[97] Brasch J., Beck-Jendroschek V., Mahn V. (2018). Photochemical Inhibition of *Trichophyton rubrum* by Different Compoundings of Curcumin. Mycoses.

[98] Slominski A.T., Slominski R.M., Raman C., Chen J.Y., Athar M., Elmets C. (2022). Neuroendocrine Signaling in the Skin with a Special Focus on the Epidermal Neuropeptides. Am. J. Physiol.-Cell Physiol..

[99] Slominski A.T., Zmijewski M.A., Plonka P.M., Szaflarski J.P., Paus R. (2018). How UV Light Touches the Brain and Endocrine System Through Skin, and Why. Endocrinology.

[100] Nogueira L., Tracey A.T., Alvim R., Reisz P., Scherz A., Coleman J.A., Kim K. (2020). Developments in Vascular-Targeted Photodynamic Therapy for Urologic Malignancies. Molecules.

[101] Beyer K., Nikfarjam F., Butting M., Meissner M., König A., Ramirez Bosca A., Kaufmann R., Heidemann D., Bernd A., Kippenberger S. (2017). Photodynamic Treatment of Oral Squamous Cell Carcinoma Cells with Low Curcumin Concentrations. J. Cancer.

[102] Abdel Fadeel D.A., Kamel R., Fadel M. (2020). PEGylated Lipid Nanocarrier for Enhancing Photodynamic Therapy of Skin Carcinoma Using Curcumin: In-Vitro/in-Vivo Studies and Histopathological Examination. Sci. Rep..

[103] Buss S., Dobra J., Goerg K., Hoffmann S., Kippenberger S., Kaufmann R., Hofmann M., Bernd A. (2013). Visible Light Is a Better Co-Inducer of Apoptosis for Curcumin-Treated Human Melanoma Cells than UVA. PLoS ONE.

[104] Niu T., Tian Y., Mei Z., Guo G. (2016). Inhibition of Autophagy Enhances Curcumin United Light Irradiation-Induced Oxidative Stress and Tumor Growth Suppression in Human Melanoma Cells. Sci. Rep..

[105] Szlasa W., Supplitt S., Drąg-Zalesińska M., Przystupski D., Kotowski K., Szewczyk A., Kasperkiewicz P., Saczko J., Kulbacka J. (2020). Effects of Curcumin Based PDT on the Viability and the Organization of Actin in Melanotic (A375) and Amelanotic Melanoma (C32)—In Vitro Studies. Biomed. Pharmacother..

[106] Roos F., Binder K., Rutz J., Maxeiner S., Bernd A., Kippenberger S., Zöller N., Chun F.K.-H., Juengel E., Blaheta R.A. (2019). The Antitumor Effect of Curcumin in Urothelial Cancer Cells Is Enhanced by Light Exposure In Vitro. Evid.-Based Complement. Altern. Med..

[107] Mani J., Fleger J., Rutz J., Maxeiner S., Bernd A., Kippenberger S., Zöller N., Chun F.K.-H., Relja B., Juengel E. (2019). Curcumin Combined with Exposure to Visible Light Blocks Bladder Cancer Cell Adhesion and Migration by an Integrin Dependent Mechanism. Eur. Rev. Med. Pharmacol. Sci..

[108] Yan G., Zhang L., Feng C., Gong R., Idiiatullina E., Huang Q., He M., Guo S., Yang F., Li Y. (2018). Blue Light Emitting Diodes Irradiation Causes Cell Death in Colorectal Cancer by Inducing ROS Production and DNA Damage. Int. J. Biochem. Cell Biol..

[109] Şueki F., Ruhi M.K., Gülsoy M. (2019). The Effect of Curcumin in Antitumor Photodynamic Therapy: In Vitro Experiments with Caco-2 and PC-3 Cancer Lines. Photodiagnosis Photodyn. Ther..

[110] Vetha B.S.S., Kim E.-M., Oh P.-S., Kim S.H., Lim S.T., Sohn M.-H., Jeong H.-J. (2019). Curcumin Encapsulated Micellar Nanoplatform for Blue Light Emitting Diode Induced Apoptosis as a New Class of Cancer Therapy. Macromol. Res..

[111] Kazantzis K.T., Koutsonikoli K., Mavroidi B., Zachariadis M., Alexiou P., Pelecanou M., Politopoulos K., Alexandratou E., Sagnou M. (2020). Curcumin Derivatives as Photosensitizers in Photodynamic Therapy: Photophysical Properties and in Vitro Studies with Prostate Cancer Cells. Photochem. Photobiol. Sci..

[112] Rutz J., Maxeiner S., Juengel E., Bernd A., Kippenberger S., Zöller N., Chun F.K.-H., Blaheta R.A. (2019). Growth and Proliferation of Renal Cell Carcinoma Cells Is Blocked by Low Curcumin Concentrations Combined with Visible Light Irradiation. Int. J. Mol. Sci..

[113] Rutz J., Maxeiner S., Justin S., Bachmeier B., Bernd A., Kippenberger S., Zöller N., Chun F.K.-H., Blaheta R.A. (2020). Low Dosed Curcumin Combined with Visible Light Exposure Inhibits Renal Cell Carcinoma Metastatic Behavior in Vitros. Cancers.

[114] Zhang C., Jiang S., Li K., Wang M., Zhu R., Sun X., Wang Q., Wang S. (2015). The Triplet State of Tanshinone I and Its Synergic Effect on the Phototherapy of Cancer Cells with Curcumin. Spectrochim. Acta A Mol. Biomol. Spectrosc..

[115] Ellerkamp V., Bortel N., Schmid E., Kirchner B., Armeanu-Ebinger S., Fuchs J. (2016). Photodynamic Therapy Potentiates the Effects of Curcumin on Pediatric Epithelial Liver Tumor Cells. Anticancer Res..

[116] Banerjee S., Prasad P., Hussain A., Khan I., Kondaiah P., Chakravarty A.R. (2012). Remarkable Photocytotoxicity of Curcumin in HeLa Cells in Visible Light and Arresting Its Degradation on Oxovanadium(Iv) Complex Formation. Chem. Commun..

[117] He G., Mu T., Yuan Y., Yang W., Zhang Y., Chen Q., Bian M., Pan Y., Xiang Q., Chen Z. (2019). Effects of Notch Signaling Pathway in Cervical Cancer by Curcumin Mediated Photodynamic Therapy and Its Possible Mechanisms in Vitro and in Vivo. J. Cancer.

[118] de Matos R.P.A., Calmon M.F., Amantino C.F., Villa L.L., Primo F.L., Tedesco A.C., Rahal P. (2018). Effect of Curcumin-Nanoemulsion Associated with Photodynamic Therapy in Cervical Carcinoma Cell Lines. BioMed Res. Int..

[119] Jiang S., Zhu R., He X., Wang J., Wang M., Qian Y., Wang S. (2016). Enhanced Photocytotoxicity of Curcumin Delivered by Solid Lipid Nanoparticles. Int. J. Nanomed..

[120] Baghdan E., Duse L., Schüer J.J., Pinnapireddy S.R., Pourasghar M., Schäfer J., Schneider M., Bakowsky U. (2019). Development of Inhalable Curcumin Loaded Nano-In-Microparticles for Bronchoscopic Photodynamic Therapy. Eur. J. Pharm. Sci..

[121] Duse L., Agel M.R., Pinnapireddy S.R., Schäfer J., Selo M.A., Ehrhardt C., Bakowsky U. (2019). Photodynamic Therapy of Ovarian Carcinoma Cells with Curcumin-Loaded Biodegradable Polymeric Nanoparticles. Pharmaceutics.

[122] Rahimi-Moghaddam F., Sattarahmady N., Azarpira N. (2018). Gold-Curcumin Nanostructure in Photothermal Therapy on Breast Cancer Cell Line: 650 and 808 Nm Diode Lasers as Light Sources. J. Biomed. Phys. Eng..

[123] Kamel A.E., Fadel M., Louis D. (2019). Curcumin-Loaded Nanostructured Lipid Carriers Prepared Using Peceol^TM^ and Olive Oil in Photodynamic Therapy: Development and Application in Breast Cancer Cell Line. Int. J. Nanomed..

[124] Machado F.C., Adum de Matos R.P., Primo F.L., Tedesco A.C., Rahal P., Calmon M.F. (2019). Effect of Curcumin-Nanoemulsion Associated with Photodynamic Therapy in Breast Adenocarcinoma Cell Line. Bioorg. Med. Chem..

[125] Khorsandi K., Hosseinzadeh R., Shahidi F.K. (2019). Photodynamic Treatment with Anionic Nanoclays Containing Curcumin on Human Triple-negative Breast Cancer Cells: Cellular and Biochemical Studies. J. Cell Biochem..

[126] Sun M., Zhang Y., He Y., Xiong M., Huang H., Pei S., Liao J., Wang Y., Shao D. (2019). Green Synthesis of Carrier-Free Curcumin Nanodrugs for Light-Activated Breast Cancer Photodynamic Therapy. Colloids Surf. B. Biointerfaces.

[127] Tsai W.-H., Yu K.-H., Huang Y.-C., Lee C.-I. (2018). EGFR-Targeted Photodynamic Therapy by Curcumin-Encapsulated Chitosan/TPP Nanoparticles. Int. J. Nanomed..

[128] Szewczyk G., Zadlo A., Sarna M., Ito S., Wakamatsu K., Sarna T. (2016). Aerobic Photoreactivity of Synthetic Eumelanins and Pheomelanins: Generation of Singlet Oxygen and Superoxide Anion. Pigment Cell Melanoma Res..

[129] Zbyradowski M., Duda M., Wisniewska-Becker A., Heriyanto, Rajwa W., Fiedor J., Cvetkovic D., Pilch M., Fiedor L. (2022). Triplet-Driven Chemical Reactivity of β-Carotene and Its Biological Implications. Nat. Commun..

[130] Makhneva Z.K., Ashikhmin A.A., Bolshakov M.A., Moskalenko A.A. (2020). Carotenoids Are Probably Involved in Singlet Oxygen Generation in the Membranes of Purple Photosynthetic Bacteria under Light Irradiation. Microbiology.

[131] Yoshii H., Yoshii Y., Asai T., Furukawa T., Takaichi S., Fujibayashi Y. (2012). Photo-Excitation of Carotenoids Causes Cytotoxicity via Singlet Oxygen Production. Biochem. Biophys. Res. Commun..

[132] Adusumilli N.C., Mordorski B., Nosanchuk J., Friedman J.M., Friedman A.J. (2021). Curcumin Nanoparticles as a Photoprotective Adjuvant. Exp. Dermatol..

